# The Host Autophagy During *Toxoplasma* Infection

**DOI:** 10.3389/fmicb.2020.589604

**Published:** 2020-10-22

**Authors:** Minmin Wu, Obed Cudjoe, Jilong Shen, Ying Chen, Jian Du

**Affiliations:** ^1^Department of Biochemistry and Molecular Biology, School of Basic Medical Sciences, Anhui Medical University, Hefei, China; ^2^The Key Laboratory of Zoonoses of Anhui, Anhui Medical University, Hefei, China; ^3^The Key Laboratory of Pathogen Biology of Anhui Province, Anhui Medical University, Hefei, China; ^4^School of Nursing, Anhui Medical University, Hefei, China

**Keywords:** *Toxoplasma gondii*, autophagy, xenophagy, LC3-associated phagocytosis, IFN-γ mediated pathogen elimination

## Abstract

Autophagy is an important homeostatic mechanism, in which lysosomes degrade and recycle cytosolic components. As a key defense mechanism against infections, autophagy is involved in the capture and elimination of intracellular parasites. However, intracellular parasites, such as *Toxoplasma gondii*, have developed several evasion mechanisms to manipulate the host cell autophagy for their growth and establish a chronic infection. This review provides an insight into the autophagy mechanism used by the host cells in the control of *T. gondii* and the host exploitation by the parasite. First, we summarize the mechanism of autophagy, xenophagy, and LC3-associated phagocytosis. Then, we illustrate the process of autophagy proteins-mediated *T. gondii* clearance. Furthermore, we discuss how the parasite blocks and exploits this process for its survival.

## Introduction

*Toxoplasma gondii* (*T. gondii*) is an obligate intracellular protozoan parasite, which can infect warm-blooded animals and cause morbidity and mortality in humans and animals worldwide (Halonen and Weiss, 2013). *T. gondii* is unified as a single species in the genus *Toxoplasma*, although recent estimates based on genomic barcoding show a much greater diversity across continents. Most strains of *Toxoplasma* isolated in Europe and North America belong to three main clonal lineages known as Types I, II, and III strains, which have different virulence in mice and may cause different human sequelae (Darde et al., 1992; Howe and Sibley, 1995; Howe et al., 1997; Machacova et al., 2016). *T. gondii* strains in South America are genetically more diverse than those in North America and Europe. However, Chinese 1 strain was reported to be the dominant strain, differing from the three main clones previously reported in East Asia, particularly in China (Zhou et al., 2010; Chen et al., 2011; Wang et al., 2013; Li et al., 2014).

*Toxoplasma gondii* is transmitted through food-borne, animal-to-human, congenital, blood transfusion, and organ transplantation (Montoya and Liesenfeld, 2004; Chu et al., 2017; Yu et al., 2017). *T. gondii* can cause human congenital and acquired infection. The congenital form may be subclinical or manifest as destructive damage to the internal organs, eyes, and brain (Halonen and Weiss, 2013; Hampton, 2015). Available data suggest about a third of the world’s population have developed long lasting serum antibodies in response to previous subclinical infection. Most of the toxoplasmosis is asymptomatic (Halonen and Weiss, 2013; Parlog et al., 2014). In rare cases, acquired toxoplasmosis could cause serious, disseminated diseases such as meningoencephalitis, pneumonia, or myocarditis. In addition, acquired ocular disease generally occurs either alone as a result of reactivation from chronic infection, or as a complication of acute systemic disease, including newly acquired disease (Montoya and Remington, 1996; Montoya and Liesenfeld, 2004; Hampton, 2015). In the acute phase of infection, tachyzoites rapidly replicate and spread to various organs through the blood or lymphatic system. In the chronic phase, *T. gondii* forms tissue cysts (bradyzoites) in which the parasite can survive for a long time or even lifetime lurking in the brain, tissue, muscle, retina, etc. (Lyons et al., 2002; Halonen and Weiss, 2013; Li et al., 2016).

Autophagy, a well-conserved and tightly regulated process in eukaryotes, which is key for cellular homeostasis, cell survival, and degradation of damaged cytoplasmic components or harmful exogenous substrates (Levine and Kroemer, 2008; Wang et al., 2009). As a key defense mechanism against infections, it also involves catching parasites inside cells and then clearing them out (Besteiro, 2017). Moreover, autophagy is thought to be an integral component of the innate immune response, targeting intracellular parasites, and parasites in damaged vacuoles and phagosomes, limiting their growth (Huang and Brumell, 2014). Recent study has shown that 4-Hydroxybenzaldehyde restricts the intracellular growth of *Toxoplasma* by activating SIRT1-mediated autophagy in macrophages (Lee et al., 2020). However, intracellular pathogens have also evolved diverse mechanisms to avoid autophagy (Steele et al., 2015). In this review, we focus on recent progress on understanding interactions between *T. gondii* and host autophagy pathways.

## Mechanisms of Autophagy

**Figure 1:**
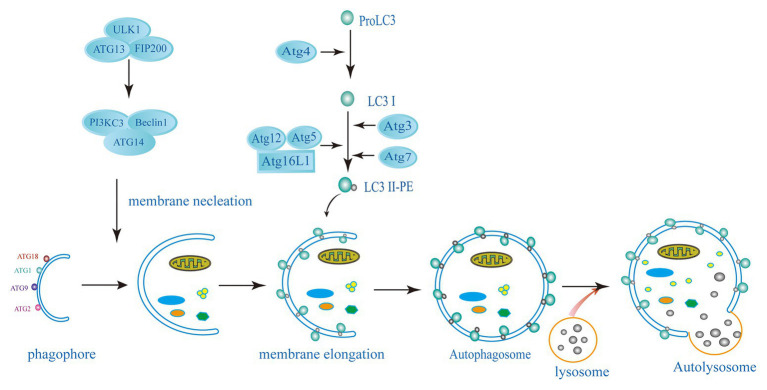
Schematic diagram of the autophagy pathway. The induction of autophagy causes the assembly and elongation of the phagophore and finally forms a structure that can surround the components in cytoplasm. The phagophore expands and develops into a double-membrane structure of autophagosomes. Targets in cytoplasm, like organelles, proteins, and microorganisms, are isolated. Autolysosome has formed by the fussing of autophagosome and lysosome, where hydrolytic enzymes in the autolysosome degrade cytoplasmic cargo. The left side shows the key proteins related to mammalian autophagy (ATG, autophagy-related: ATG3, ATG5, ATG7, ATG4, ATG12, ATG13, ATG14, ATG16L1, FIP200, ULK1, PI3KC3, Beclin1, LC3, and PE).

Autophagy is a conservative catabolism system in cells, in which double-membraned autophagosome engulfs cytoplasmic components and degrades them after fusion with lysosomes (Choi et al., 2013; Paulus and Xavier, 2015; Galluzzi et al., 2017). Autophagy is mainly dependent on autophagy (ATG) proteins leading to the formation of autophagosome and lysosomal degradation of autophagy substrates. This pathway involves several different steps, such as the formation of a cup-shaped double membrane capable of engulfing specific substances or chunks of cytoplasm; membrane elongation and closure to form autophagosomes; transport and fusion of autophagosomes and lysosomes; and finally, degradation of the enclosed cytoplasmic contents within the autophagosome and nutrient recycling (Nakatogawa et al., 2009; Parzych and Klionsky, 2014; Figure 1). Starvation and the mammalian target of rapamycin (mTOR) kinase inhibition induce autophagy to provide nutrients for the basic physiological processes of the cells and to maintain homeostasis. Nutrient deprived conditions induce autophagosome formation *via* the mTORcomplex 1 (mTORC1) inhibition in mammalian cells. The initiation complex of ULK1 activates the phosphatidylinositol 3-kinase (PI3K) complex of Beclin1-Vps34-Vps15-Atg14L (Deretic, 2011; Choi et al., 2014; Paulus and Xavier, 2015). The expansion or elongation of the phagophore need the involvement of two ubiquitin-like (UBL) conjugation systems. The first UBL conjugation system involves the formation of Atg12-Atg5-Atg16 complex. In yeast cells, Atg12 binds covalently to Atg5. This Atg12-Atg5 conjugation is highly dependent on Atg7 (E1 activating enzyme) and Atg10 (E2 activating enzyme). Then, Atg16 non-covalently binds to Atg5 forming the Atg12-Atg5-Atg16 complex, following the Atg12-Atg5 conjugation. The second UBL conjugation system involves the Atg8 or LC3 (in mammals) system. Atg8/LC3 is activated by the EI-like enzyme Atg7 and transferred to the E2-like enzyme Atg3. In the final step, phosphatidylethanolamine (PE) binds to the C-terminal glycine Atg8 to form LC3-phosphatidylethanolamine conjugate (LC3-II) and incorporated into the autophagosomal membrane (Hwang et al., 2012; Huang and Brumell, 2014; Biering et al., 2017; Choi et al., 2017; Wacker et al., 2017). The lysosome subsequently fuses with the outer membrane of the autophagosome to deliver their cytoplasmic cargo for degradation and recycling (Melzer et al., 2008; Paulus and Xavier, 2015; Selleck et al., 2015). Cytoplasmic cargoes are captured in double-membrane structure, called “autophagosomes,” which then mature into autolysosomes that degrade or otherwise eliminate captured cargoes. Another strategy employed by host cells to capture and degrade invading intracellular pathogens is xenophagy. It is a key defense mechanism against various infections involving the elimination intracellular pathogens such as protozoans, bacteria, and viruses (Mizushima et al., 2011; Paulus and Xavier, 2015; Galluzzi et al., 2017). Xenophagy is also considered as an innate component of cellular immune response.

Autophagy proteins may be involved in certain cellular pathways leading to the elimination of intracellular pathogens but do not constitute autophagy. These cellular pathways are established under certain cellular conditions; some bypass proteins involved in elongation and closure (Atg7, Atg5, and LC3), while others bypass proteins necessary for initiation (ULK1) and nucleation (Beclin1; Steele et al., 2015; Galluzzi et al., 2017). LC3-associated phagocytosis (LAP) is an example of such a process, involves the recruitment of LC3 to single-membrane phagosomes, which surround intracellular pathogens or dead cells (Paulus and Xavier, 2015). This process is independent of the ULK1 initiation complex, while the PtdIns3K complex is important for LAP initiation (Martinez et al., 2015; Besteiro, 2019). During PAMP receptor activation, PI3PK complex is recruited to the phagosomal membrane (Ma et al., 2012). This complex lacks ATG14 but is composed of Rubicon and UV resistance-associated gene (UVRAG; Martinez et al., 2015). With Rubicon’s help, NADPH oxidase 2 (NOX2) then enters the phagosomes. Activation of NOX2 and PI3PK leads to the production reactive oxygen species (ROS) and initiate lipidation of the phagosomal membrane, respectively, and ultimately leading to the formation of LAPosome (LC3-decorated phagosome; Ueyama et al., 2011). Fusing with lysosomes to form a lytic compartment, the LAPosomes then degrades the cargo. Although the effect of LC3 on LAPosome is still unknown, some studies suggested that LC3 could accelerate its maturation by fusion with lysosomes (Martinez et al., 2015; Besteiro, 2019).

## Mechanisms of Host Autophagy Proteins-Mediated *T. Gondii* Clearance

*Toxoplasma gondii* replicate in the host cell by forming a special endocytic vacuole, called parasitophorous vacuole (PV). The membrane of PV allows *T. gondii* to develop while protected from intracellular cytoplasmic defense mechanisms (Orlofsky, 2009; Portillo et al., 2017). Increasing literature indicates that *T. gondii* actively induces autophagic pathways in the infected host cells (Orlofsky, 2009; Wang et al., 2009; Chu et al., 2017; Saeij and Frickel, 2017; Subauste, 2019). Two types of immune response are required in the control of *T. gondii*: clearance of *T. gondii* by CD40-mediated autophagy and IFN-γ-induced the clearance of *T. gondii* through autophagy proteins. Both of them are essential for killing parasites within host cells.

### Clearance of *T. gondii* by CD40-Mediated Autophagy

The parasitic vacuole is formed during the active invasion of *T. gondii*. Vacuolar membranes have been extensively modified by removing many proteins from host cells and inserting parasite-derived proteins into parasitic vacuoles. Once the PV is formed, its nonfusion properties will be unchanged (Subauste, 2009). So, whether there is a way to change the nonfusion properties of PV is the key question in the interaction between *T. gondii* and the immune system. Previous studies have demonstrated that this can be achieved *via* stimulation of CD40 (Andrade et al., 2006; Subauste et al., 2007b). In macrophages infected with *T. gondii*, stimulation with CD40 can induce the fusion of the PV with lysosomes in an autophagy-dependent manner.

**Figure 2:**
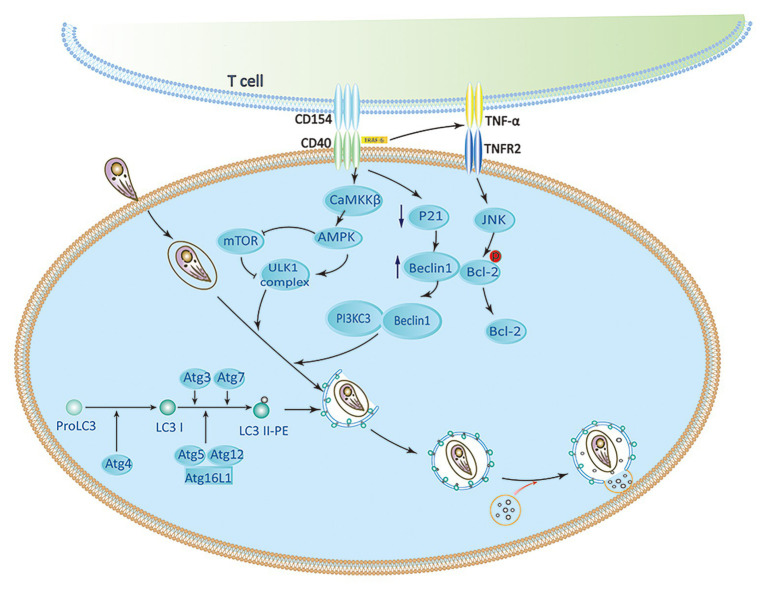
CD40 induces activation of autophagy signaling pathway. CaMKKβ-mediated Threonine-172 AMP-activated protein kinase (AMPK) phosphorylation can be induced by CD40, which leads to Serine-555 ULK1 phosphorylation and ULK1-mediated autophagy. CD40 may upregulate Beclin 1 protein levels by reducing p21. The accumulation of TRAF6 in the cytoplasmic tail of CD40 enhances TNF-α autocrine production, which leads to the phosphorylation of Bcl-2 at Serine 87 in JNK-dependent way and separation of Bcl-2 from Beclin1. ULK1, Beclin 1, PI3KC3, ATG5, ATG7, and lysosomal enzymes play an important role in killing of *Toxoplasma gondii* caused by CD40 (Subauste, 2009; Liu et al., 2016).

As a member of the TNF receptor superfamily, CD40 expresses on various nonhematopoietic cells and APCs (Subauste, 2009; Van Grol et al., 2013). Several studies have demonstrated that CD40 triggers signaling pathways, which are upstream of ULK1 and Beclin1-PI3KC3 complexes to activate autophagy. CD40 stimulates autophagy *via* pathways involving CaMKKβ, AMP-activated protein kinase (AMPK), and ULK1, leading to kill *T. gondii* (Liu et al., 2016). The phosphorylation of Ser-555 ULK1 and autophagy mediated by ULK1 are caused by AMPK (Russell et al., 2013). CD40 can induce phosphorylation of Thr-172 AMPK mediated by CaMKKβ. Besides direct effects on the ULK1/2 complex, AMPK also inhibit mTORC1 by phosphorylating Raptor (a mTORC1 binding partner). CD40 also interacts with TRAF6 to stimulate TNF-α production in macrophages. Recruitment of TRAF6 in the cytoplasmic tail of CD40 enhances the expression of TNF-α. It causes phosphorylation of Bcl-2 at Ser-87 in JNK-dependent way, and binding Beclin 1 to PI3KC3 to induce autophagy (Subauste et al., 2007a; Liu et al., 2016). CD40 can also induce the death of *T. gondii* by lowering the level of P21 and upregulating the autophagy molecule Beclin 1 (Portillo et al., 2010; Figure 2). CD40 signaling triggers pathogen-lysosome fusion *via* the autophagic mechanism. ULK1/2 complexes and the Beclin1-PI3KC3 complex activation promote the formation and maturation of autophagosome by recruiting Atg proteins to the isolation membrane (Liu et al., 2016). The phagophore is produced by polymerization of Atg8 (LC3) and Atg12-Atg5 UBL conjugation systems (Kihara et al., 2001; Ohsumi, 2001). The isolation membrane envelops organelles and cytosol, which leads to forming the autophagosomes. Therefore, autophagosomes are recruited around the PV and fused with late endosomal lysosomes to kill *T. gondii* (Zhao et al., 2007; Subauste, 2009; Chu et al., 2017).

### IFN-γ Restricts *T. gondii* Through Autophagy-Independent Effects of Autophagy Proteins

**Figure 3:**
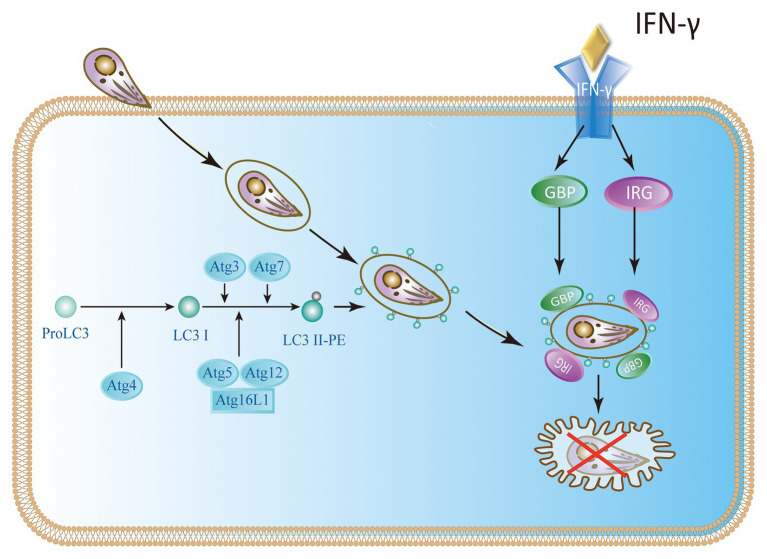
IFN-γ restricts the growth of *T. gondii* in mouse cells. In mice, the Atg proteins Atg7, Atg3, and the Atg12-Atg5-Atg16L1 complex play a key role in the delivery and binding of LC3 to the autophagosome membrane. They are also involved in targeting immunity-related p47GTPases (IRG) and guanylate-binding protein (GBP) to *Toxoplasma* parasitophorous vacuole membrane (PVM).

To restrict the infection, IFN-γ-stimulated murine cells are armed with effector molecules, such as the immunity-related p47GTPases (IRGs) and guanylate-binding proteins (GBPs; Feng et al., 2008). Studies have revealed that the LC3 conjugation system is necessary to recruit IRGs and GBPs to the parasitophorous vacuole membrane (PVM; Choi et al., 2014; Haldar et al., 2014; Ohshima et al., 2014; Lee et al., 2015). Atg5 is an important autophagic gene and is particularly useful for targeting IRGs and GBPs to PVM containing *T. gondii*. Without ATG5, IRGs and GBPs are usually induced by IFN-γ, and they form aggregates in the cytoplasm instead of targeting vacuoles containing pathogens (Zhao et al., 2008; Choi et al., 2014, 2017; Biering et al., 2017). Once these GTPases are recruited to the LC3-tagged PVM, the PVM surrounding parasites shows extensive vesiculation and blebbing with clusters of small vesicles in the purlieu of the vacuole. At these sites, IRGs localize to small vesicular forms with dense coats from the adjacent PVM, stripping the PV membrane of *T. gondii*. Denuded parasites are enveloped in autophagic vacuoles that eventually fuse with lysosomes and ultimately limit the growth of *T. gondii* growth (Ling et al., 2006; Zhao et al., 2008; Clough et al., 2016; Coppens, 2017). PVMs of type I virulent strains are much less intensely loaded with IRG proteins than those of type II or type III avirulent strains in single infection or co-infections (Khaminets et al., 2010). Although IRGs and GBPs participate in the disruption of parasite PVM, the molecular mechanisms of how these IFN-γ induced GTPases interact with the autophagic machinery are poorly understood (Melzer et al., 2008; Whitmarsh and Hunter, 2008; Choi et al., 2014; Biering et al., 2017; Figure 3).

**Figure 4:**
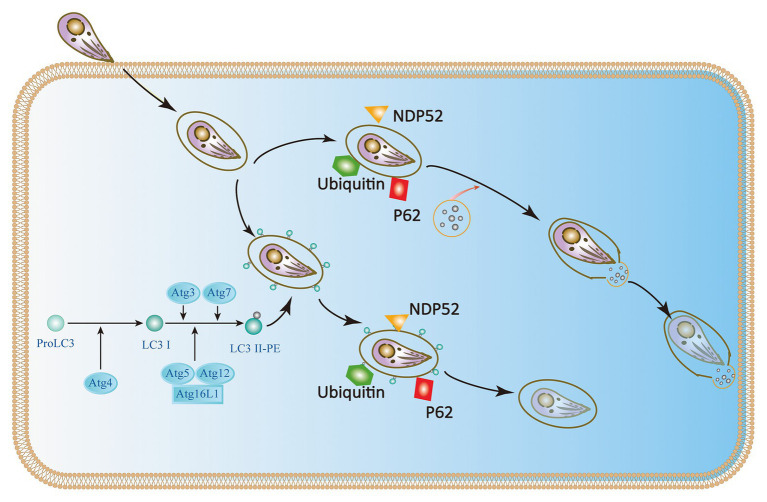
IFN-γ restricts the growth and proliferation of *T. gondii* in human cells. In HeLa cells, the Atg proteins Atg7, Atg3, and the Atg12-Atg5-Atg16L1 complex target ubiquitin to the *Toxoplasma* PVM, which causes the recruitment of p62 and NDP52 and subsequently LC3. The parasite is encapsulated in a double-membrane autophagy structure and cannot grow and replicate further. In human umbilical vein endothelial cells (HUVEC) cells, *T. gondii* encapsulated in parasitophorous vacuole (PV) is recognized by host cell immune effector factors (K63Ub, p62, and NDP52), which leads to lysosomal fusion and subsequent parasite destruction.

Although some studies have identified p47 IRGs as the primary IFN-γ-induced mechanism of anti-*Toxoplasma* infection in mice during the acute phase, there is a lack of multitude of IRGs in humans (Subauste, 2009; Clough et al., 2016; Krishnamurthy et al., 2017). Immunity-related GTPase family M protein (IRGM) and immunity-related GTPase cinema (IRGC) are the only two human IRGs. But neither of them are induced by IFN-γ. IRGM plays a role in autophagy and host resistance against *Mycobacterium tuberculosis*; but its role in toxoplasmosis has not been determined (Portillo et al., 2010; Deretic, 2011; Niedelman et al., 2013; Krishnamurthy et al., 2017). Furthermore, seven IFN-γ-inducible Guanylate binding proteins (GBPs) expressed in humans are involved in IFN-γ dependent clearance of pathogens such as *Chlamydia* and viruses. There is currently no evidence of GBP-mediated inhibition of *T. gondii* in humans (Ohshima et al., 2014). Selleck et al. identified new roles for ubiquitination and recruitment of autophagic adapters p62 and NDP52, which could control *T. gondii* replication in IFN-γ activated human cells (Choi et al., 2014; Lee et al., 2015; Selleck et al., 2015; Clough et al., 2016). The IFN-γ mediated ubiquitin-driven restriction pathways of *Toxoplasma* type II varies with cell type. Ubiquitin-targeted PVs found in HeLa cells do not acidify but instead restrict *Toxoplasma* type II by stunting growth. However, IFN-γ stimulated human umbilical vein endothelial cells (HUVEC) have been shown to restrict *Toxoplasma* in a manner independent of autophagy, where HUVEC exhibits vacuolar lysosomal acidification and subsequent parasite destruction. Thus, we conclude that the two different human cell types deploy the same initial defense molecules ubiquitin, p62 and NDP52 to similar numbers but diverge in the ultimate strategy (Clough et al., 2016; Saeij and Frickel, 2017). Neither of these mechanisms works in all cell types, suggesting the IFN-γ mediated control of *T. gondii* exists multiple overlapping pathways in human cells (Niedelman et al., 2013; Selleck et al., 2015; Bando et al., 2018). In addition, ATG16L1 and ATG7 are required to encapsulate PV in multiple host membranes. They do not destroy vacuoles or transmit parasite to lysosomes, but they limit nutrient absorption and inhibit parasite growth (Choi et al., 2014; Lee et al., 2015; Selleck et al., 2015). Therefore, IFN-γ mainly relies on ubiquitination and core autophagy to mediate membrane engulfment and cell growth restriction in human cells (Figure 4).

## Host Cell Signaling Is Manipulated by *Toxoplasma Gondii* to Avoid Autophagy Targeting

Autophagy is mainly a process of sustaining life. It could not only coordinate the degradation and circulation of important macromolecules such as lipids and amino acids response to stress but also contribute to catching the intracellular parasites and transporting them for destruction (Bando et al., 2018). Avoiding lysosomal degradation is critical for the survival of *T. gondii*. *Toxoplasma* can interfere with the host autophagy machinery to escape targeting or even promote their intracellular growth by exploiting autophagy components (Wang et al., 2009; Deretic, 2011; Ohshima et al., 2014). Currently, it is still unclear how *Toxoplasma* interferes with autophagy.

**Figure 5:**
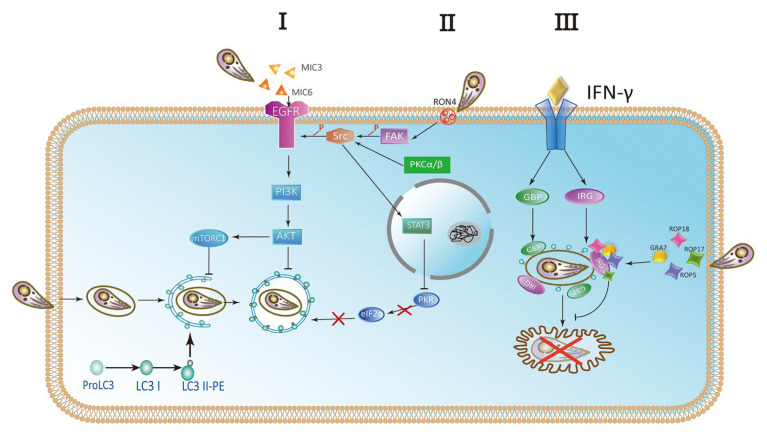
Parasites escape host autophagy destruction. (I) A hypothetical model of *Toxoplasma* controlling host autophagy by the epidermal growth factor receptor (EGFR)-Akt signaling pathway. MIC protein is released from micronemes upon invasion. MIC3 and MIC6 bind to the host EGFR through the EGF-like domain and are activated by phosphorylation. Phosphatidylinositol 3-kinase (PI3K)-induced Akt phosphorylation and Akt-dependent activation of mTORC1 block the autophagy pathway and signify *T. gondii* to survive. (II) During the invasion of host cells, RON4 expression was associated with focal adhesion kinase (FAK) activation. In turn, FAK activates Src leading to the transactivation of EGFR in a Src-dependent way. Furthermore, the STAT3 signaling pathway is facilitated to prevent PKR and eIF2a from activation. Blocking the signaling causes the activation of PKR and eIF2a, resulting in autophagic targeting and the killing of parasite. At later stages of the intracellular stage of *T. gondii*, autophosphorylation of EGFR is maintained through PKCα/PKCβ-Src signaling. (III) The complex of GRA7 and ROP18-ROP17-ROP5 phosphorylates IFN-γ-activated IRGs and GBPs permanently inactivates them to ensure the integrity of PVM and is conducive to the survival of parasite.

*Toxoplasma gondii* activates host cell signaling cascade of the epidermal growth factor receptor (EGFR) by PI3K to avoid targeting autophagy mechanisms to parasitic vacuoles (Muniz-Feliciano et al., 2013; Coppens, 2017; Portillo et al., 2017). Parasites can induce the phosphorylation of EGFR in host cells by secreting several proteins containing multiple domains homologous to EGFR, such as MIC3 and MIC6. PI3K-mediated Akt phosphorylation can activate the autophagosome negative regulator mTORC1, which leads to reverse regulation of autophagy (Muniz-Feliciano et al., 2013; Coppens, 2017; Lopez Corcino et al., 2019). Thus, the Akt signaling pathway is critical for escaping host autophagy to promote parasite survival (Figure 5 I). The moving junction is characterized by the expression of RON4. The formation of the moving junction is accompanied by the activation of focal adhesion kinase (FAK) in the mammalian cell during the invasion. The activation of Src depends on FAK, and then causes the transactivation of EGFR. The activation of EGFR recruits STAT3 signaling to block the activation of key stimulators of autophagy, PKR, and eIF2α. They prevent *T. gondii* from becoming a target (Portillo et al., 2017). At the later stage of the intracellular phase of *T. gondii*, EGFR autophosphorylation is maintained through prolonged PKCα/PKCβ-Src signaling, which in turn promote the survival of *Toxoplasma* through Akt (Lopez Corcino et al., 2019; Figure 5 II).

IFN-γ is a main effector for activating mammalian cell responses to *T. gondii*. Recruitment and loading of effector IRGs on PVM are induced by IFN-γ (Martens et al., 2005; Ling et al., 2006; Khaminets et al., 2010). The deposition of ubiquitin on the PVM is promoted by IRGs and subsequently the accumulation of p62-dependent GBP (Haldar et al., 2015). In mouse macrophages and fibroblasts activated by IFN-γ, LC3 is recruited to PVM and can target IRGs to PVM membrane (Park et al., 2016). Associating with the small GTPase ADP-ribosylation factor 1 (Arf1), Gate-16 mediates IRG recruitment (Sasai et al., 2017). Recruitment of IFN-γ-activated IRGs and GBPs resulted in the vesicle formation and rupture of PVM, resulting in the release of *T. gondii* into the cytoplasm and the death of *T. gondii* in mouse cells (Martens et al., 2005; Zhao et al., 2008, 2009; Choi et al., 2014; Ohshima et al., 2014). Rop18 is a rhoptry protein, also a polymorphic protein kinase. It mainly determines the virulence of parasite in mice (Taylor et al., 2006; Lei et al., 2014). Together with ROP17, ROP18 complexes mediate the protection from the IRG pathway. The phosphorylation ability of ROP18-ROP17 depends on the presence of virulent alleles of pseudokinase ROP5 (Behnke et al., 2011; Reese et al., 2011). GRA7 is a dense granule protein. It is also a part of the complex of ROP18-ROP17-ROP5 (Hermanns et al., 2016). Binding a conserved surface of IRG, ROP5 proteins promote IRG remain in an inactive GDP binding conformation. As a result, GTP-dependent activation of IRG is prevented. Simultaneously, threonines in the nucleotide-binding domain are exposed. Then ROP18 and ROP17 kinases phosphorylate threonines, resulting in permanent inactivation of IRG (Fleckenstein et al., 2012; Reese et al., 2014; Figure 5 III).

## Concluding Remarks

In summary, autophagy is critical in maintaining cellular homeostasis and plays a key role in mechanism against a large number of intracellular pathogens including *T. gondii* as this pathway can degrade intracellular pathogens *via* autophagolysosomes. The interaction between *T. gondii* and host autophagy is a mutual process. However, *T. gondii* has developed complex evolutionary adaptation and evasion mechanisms to avoid host cell phagocytic recognition. Some PVM associated proteins (rhoptries and dense granules) are known to phosphorylate IRGs, inactivating IRGs. In recent years, the mechanism of *T. gondii* recognition and invasion has been extensively investigated. However, the mechanisms by which parasites antagonize these responses in different cell lines are still elusive. For instance, in mouse cells, IRGs contribute to immune control of *T. gondii*, but how it contributes to parasite control in human cells remains enigmatic. Understanding the mechanisms by which these parasites prevent the host’s innate immune defenses and escape autophagy may provide the basis to design new therapeutic strategies to treat toxoplasmosis.

## Author Contributions

JD designed the work. MW and OC wrote the first draft of the manuscript. JS and YC did critical revision of the article. All authors contributed to the article and approved the submitted version.

### Conflict of Interest

The authors declare that the research was conducted in the absence of any commercial or financial relationships that could be construed as a potential conflict of interest.
